# Recombinant Expression and Characterization of an Alkali-tolerant UDP-Glycosyltransferase from *Solanum lycopersicum* and Its Biosynthesis of Gastrodin

**DOI:** 10.4014/jmb.2410.10029

**Published:** 2024-11-22

**Authors:** Lin Ge, Wenxin Xu, Ruobing Jia, Yu Xia

**Affiliations:** 1 College of Biopharmacy, Suzhou Chien-Shiung Institute of Technology, 1 Jian Xiong Road, Taicang 215411, P.R. China; 2 Jiangsu Provincial Novel Anti-Tumor Targeted Drug Conjugate Engineering Research Center, Suzhou 215411, P.R. China

**Keywords:** Gastrodin, UDP-glycosyltransferase, *Solanum lycopersicum*, biosynthesis, alkali-tolerant

## Abstract

Gastrodin is the main bioactive component of *Gastrodia elata*, which has many excellent pharmacological activities. In this study, UDP-glycosyltransferase from *Solanum lycopersicum* (SlyUGT) was overexpressed, purified and characterized, and it can be used for biosynthesis of gastrodin. The SlyUGT maximum enzyme activity was 29.48 mU/ml, and its relative molecular weight was about 78.5 kDa. The SlyUGT was purified 16.1-fold by GST affinity resin with an overall recovery of 53.9% and specific activity of 20.9 mU/mg. The optimal temperature of SlyUGT was 40°C, and it exhibited excellent thermal stability at 35°C and 40°C. Furthermore, the SlyUGT had the highest activity at pH 9.5 and good pH stability at pH 5.5-10.5. The enzyme can tolerate low concentrations of DMSO and UDP. In addition, the values of *K*_M_ and *V*_max_ were found to be 0.65 mM and 74.60 mU/mg respectively. The SlyUGT could convert *p*HBA into gastrodin by using UDP-glucose as a sugar donor. Under the optimal biosynthesis conditions, the gastrodin production reached 559.83 mg/l, and the corresponding molar conversion rate reached 97.82%. The results showed that SlyUGT has potential application value in the preparation of gastrodin.

## Introduction

Gastrodin is the main bioactive component of the precious herb *Gastrodia elata*, a phenolic glycoside that is chemically known as 4-hydroxybenzyl alcohol-4-O-β-D-glucopyranoside, which has a good clinical effect on cardiovascular diseases [1], and is also widely used in the adjuvant treatment of vertigo, neuralgia, headache, neurasthenia and epilepsy [2], with remarkable curative effect and no obvious toxic and side effects [3]. In addition, gastrodin has anti-inflammatory [4], anti-anxiety [5], anti-alcoholic liver injury [6], anti-oxidation [7], anti-obesity, neuroprotection and memory improvement [8]. At present, there are as many as 44 kinds of drugs and health products on the market containing gastrodin as the main component [9]. Up to now, gastrodin production mainly adopts plant extraction method and compound synthesis method [3, 10]. However, wild gastrodia elata resources are scarce and artificial cultivation is complicated [11], and the content of gastrodin in plant gastrodia elata is extremely low (mass fraction < 0.7%) [9], resulting in high extraction cost and long extraction time. Due to the complex structure of gastrodin, chemical synthesis of gastrodin produces many analogues, which is challenging to separate. Moreover, the toxic phenols, phosphates and bromides used in the production process can lead to serious environmental pollution [12]. Biological method has the advantages of strong specificity, mild reaction conditions and low pollution. Therefore, in recent years, there have been a lot of reports on the preparation of gastrodin by biological method [9, 10, 12, 13], which has become a research hotspot.

Glycosyltransferases can take small molecular weight compounds as receptor substrates and uridine 5'-diphosphate (UDP) as donors [14]. The research report shows that gastrodin can be biosynthesized by glycosylation of *p*-hydroxybenzyl alcohol by UDP-glycosyltransferase [13]. Therefore, the catalytic activity of UGT plays a decisive role in the biosynthesis of gastrodin. However, glycosyltransferase used for biosynthesis of gastrodin in the metabolic pathway of *Gastrodia elata* is unknown. Although it has been reported that a single gene encoding glycosyltransferase may participate in the natural biosynthesis pathway of gastrodin [11], the sequence of natural UGT used for gastrodin biosynthesis is still unclear [12, 13]. In this study, UDP-glycosyltransferase from *Solanum lycopersicum* (SlyUGT) was overexpressed in *E. coli* BL21(DE3), and the expression conditions of the enzyme SlyUGT were optimized. Meanwhile, its enzymatic characteristics were determined with *p*HBA as glycosyl receptor and UDP-glucose as glycosyl donor. In addition, the biosynthesis conditions of gastrodin by SlyUGT were also studied. The results suggested that SlyUGT had potential application value in gastrodin biosynthesis.

## Materials and Methods

### Materials

Plasmid pGEX-2T was obtained from GE Healthcare, which was employed as expression vector. *E. coli* strain BL21(DE3) competent cell was obtained from Beijing TransGen Biotech Co. Ltd., (China) which was used for protein overexpression. Luria-Bertani (LB) medium was used to grow *E. coli* cells for protein expression and supplemented with antibiotics when required.

The modified Bradford protein assay kit and dimethyl sulfoxide (DMSO) were obtained from Shanghai Beyotime Biotech Co. Ltd., (China) Gastrodin, *p*-hydroxybenzyl alcohol (*p*HBA), UDP-glucose, UDP, and isopropyl-β-D-thiogalactopyranoside (IPTG) were obtained from Shanghai Aladdin Biotech Co. Ltd., The GSTSep Glutathione Agarose Resin GST, Gravity Chromatography Columns, and reduced glutathione (GSH) were obtained from Shanghai Yeasen Biotech Co. Ltd., (China) The Protein Marker, restriction endonucleases BamH I and EcoR I were obtained from Beijing TransGen Biotech Co. Ltd., (China)

### Plasmid Constructions

*SlyUGT* from *Solanum lycopersicum* (GenBank No. NP_001307116.1) was synthesized to incorporate the *E. coli* codon in Suzhou GENEWIZ Biotech Co. Ltd. The synthesized genes *SlyUGT* was inserted into the expression vector pGEX-2T by restriction endonucleases BamH I and EcoR I to create the recombinant plasmid pGEX-SlyUGT.

### Optimizing Expression Conditions of Recombinant Enzyme SlyUGT

Firstly, the recombinant expression vector pGEX-SlyUGT was transformed into *E. coli* BL21(DE3) competent cells by heat shock transformation, and the positive transformants were screened by ampicillin resistance. Then, the positive recombinant transformants were inoculated under sterile conditions into 6 ml LB medium containing ampicillin and cultured at 37°C for 12 h. Finally, the seed liquid was inoculated into 250 ml fresh LB medium containing ampicillin antibiotic with 1% inoculum for further cultivation, and IPTG was added to induce expression under appropriate conditions. The effects of different induction temperatures (20°C, 25°C, 30°C, 37°C), inducer IPTG concentrations (0, 0.05, 0.1, 0.2, 0.3, 0.4 mM), induction OD600s (0.8, 1.0, 1.2, 1.5, 1.8, 2.0), and induction times (12, 18, 24, 36, 42, 48 h) on enzyme yield were studied.

### Purification and Concentration Determination of Recombinant Enzyme SlyUGT

The recombinant *E. coli* cells cultured under the optimal expression and induction conditions were collected by centrifugation, then clarified and resuspended in PBS buffer (pH 7.4), and finally the cells were broken by ultrasonic crusher. The resulting supernatants were loaded on GSTSep Glutathione Agarose Resin GST affinity column and eluted with elution buffer (pH 7.4 PBS buffer containing 10 mM glutathione [GSH]). The resulting proteins were examined by SDS-PAGE and analyzed by a gel imager.

Protein concentrations were assayed according to the Bradford method by using a Bradford protein assay kit with bovine serum albumin as the standard protein. The protein concentration was measured at 595 nm [15].

### Activity of Recombinant Enzyme SlyUGT

SlyUGT activity was assessed using *p*HBA as a substrate. The reaction volume is 100 μl, including 1 mM *p*HBA, 50 mM glycine buffer (pH 9.5), 1 mM UDP-glucose, and suitable amount of purified SlyUGT. The reaction was terminated after 1 h at 40°C by adding 4 μl of 10% trifluoroacetate water and 900 μl methanol. Then, the reaction products were detected by high-performance liquid chromatography (HPLC). One unit of enzyme activity was defined as the amount of enzyme necessary to liberate 1 μmol of gastrodin of per min under the assay conditions.

### Characterization of Recombinant Enzyme SlyUGT

The optimum pH for SlyUGT was measured with 50 mM citrate-phosphate buffer (5.0, 5.5, 6.0, 6.5, 7.0, 7.5, 8.0), PB buffer (6.0, 6.5, 7.0, 7.5, 8.0), Tris-HCl (7.0, 7.5, 8.0, 8.5, 9.0, 9.5), and glycine buffer (8.5, 9.0, 9.5, 10.0, 10.5) at 40°C for 1 h respectively, and the pH corresponding to the highest enzyme activity was the optimal pH.

The optimum temperature for the enzyme activity was measured at 25°C, 30°C, 35°C, 40°C, 45°C, and 50°C at optimal pH for 1 h, and the temperature corresponding to the highest enzyme activity was the optimal temperature.

The thermal stability of SlyUGT was determined by measuring the inactivation rate of SlyUGT enzyme at 35°C, 40°C and 45°C when the pH value of the glycine buffer was 9.5.

The pH stability of SlyUGT was determined by mixing the enzyme solution and buffer solution with the above four different pH buffers in a ratio of 1:1, then keeping the temperature at 35°C for 4 h, and finally determining the residual enzyme activity of SlyUGT.

Common metal ions, and chemical reagents were selected to determine the effect on enzyme activity, and their final concentrations in the reaction system were controlled to be 1 mM. The relative activity of enzyme was determined by comparing with control.

### Determination of Kinetic Parameters

The kinetic parameters of SlyUGT, K_M_, and V_max_, were measured from Michaelis-Menten plots by determining the initial reaction rates with different *p*HBA concentrations (0.2 mM, 0.4 mM, 0.5 mM, 1 mM, 2 mM, 3 mM, 4 mM) at pH 9.5 and 40°C for 1 h.

### Effects of DMSO and UDP Concentration on the SlyUGT Activity

To determine the effects of DMSO and UDP on the SlyUGT activity, different concentrations of DMSO (1%, 2%, 3%, 5%, 6%, 8%, 10%, 12%) and UDP (0.25, 0.5, 1.0, 1.5, 2.0, 2.5, 3.0 mM) were added into the reaction and the SlyUGT activity was measured.

### Optimization of Reaction Conditions for Gastrodin Biosynthesis in Vitro

The reaction system was 100 μl, which contained *p*HBA, UDP-glucose, SlyUGT, and glycine buffer. The reaction was carried out in a metal bath. The biosynthesis conditions of gastrodin were optimized., including different pH value of glycine buffer (8.5, 9.0, 9.5, 10.0, 10.5), temperature (25°C, 30°C, 35°C, 40°C, 45°C), ), UDP-glucose concentration (1 mM, 2 mM, 3 mM, 4 mM, 5 mM, 6 mM, 7 mM, 8 mM, 9 mM, 10 mM) , SlyUGT concentration (2 mU/ml, 4 mU/ml, 6 mU/ml, 8 mU/ml, 10 mU/ml, 12 mU/ml, 14 mU/ml, 16 mU/ml, 18 mU/ml), and reaction time (0 h, 0.25 h, 0.5 h, 1 h, 2 h, 3 h, 4 h, 5 h, 6 h, 7 h, 8 h). The reaction was started by adding the purified SlyUGT. The reacted samples were stopped by adding 4 μl of 10% trifluoroacetate water and 900 μl methanol.

### HPLC Analysis

HPLC analysis of *p*HBA and gastrodin was performed using an Agilent HPLC 1200 system and an Agela Innoval C18 (4.6 × 250 mm; i.d., 5 μm) column. The mobile phase A and mobile phase B used in HPLC are methanol and 0.1% trifluoroacetic acid water, respectively. The HPLC conditions were as follows: 0-6 min 10%solvent A and 90% solvent B, 6-21 min 90% solvent A and 10% solvent B, 21-25 min 90% solvent A and 10% solvent B, 25-30 min 10% solvent A and 90% solvent B, 30-35 min 10% solvent A and 90% solvent B. The flow rate was 1.0 ml/min, the column was maintained at 30°C, and detection was performed by monitoring the absorbance at 225 nm.

### Statistical Analysis

The data were expressed as means ± standard deviation (SD) and were analyzed using Student’s *t*-test to determine any significant differences. All statistical analyses were conducted using SPSS 10.0 statistical software. Cases in which *p* values of < 0.01 were considered statistically significant.

## Results and Discussion

### Optimization of Culture Conditions

**Fig. 1:**
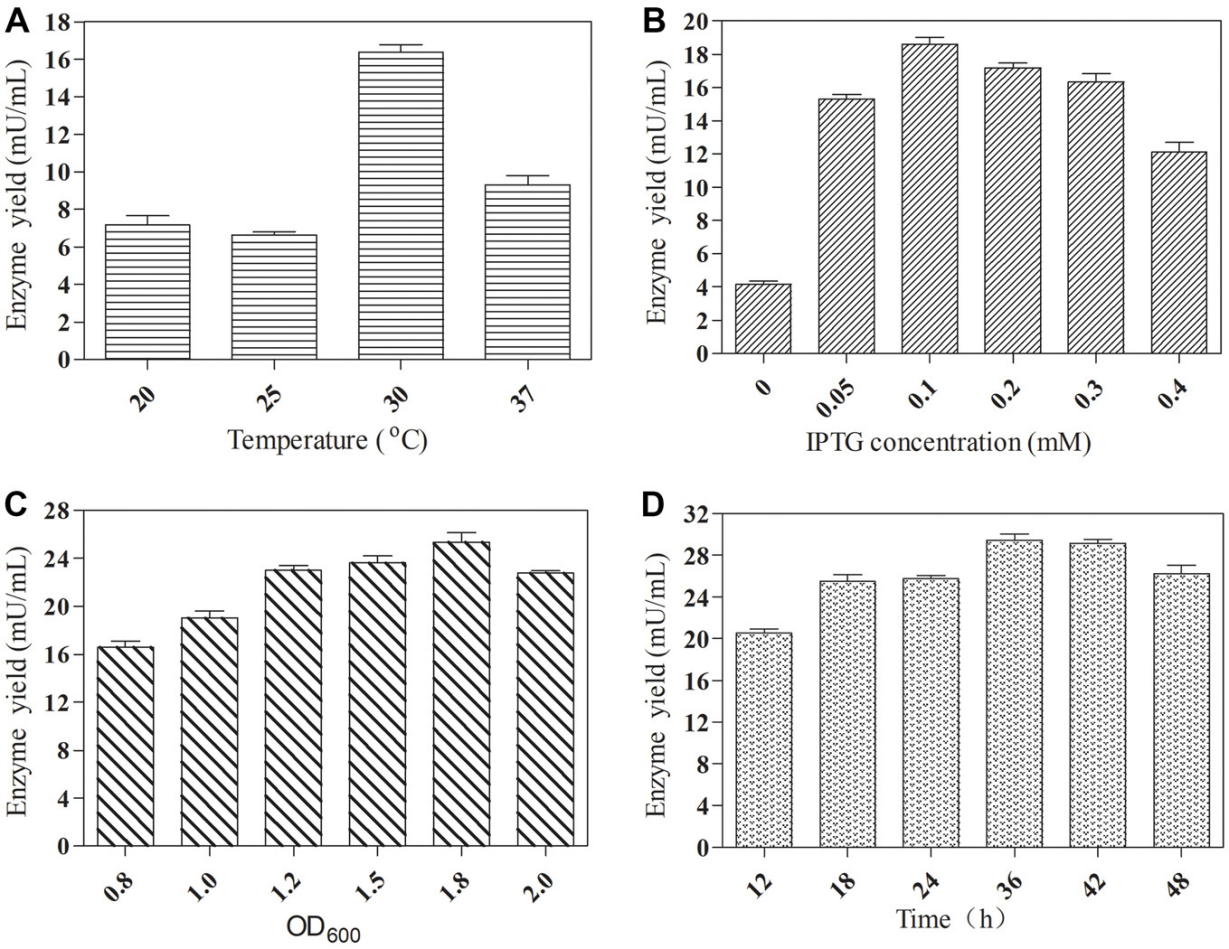
Optimization of culture conditions of SlyUGT: (**A**) induction temperature; (**B**) IPTG concentration; (**C**) OD600; (**D**) induction time. Data represent the means of three experiments, and error bars represent the standard deviation.

**Fig. 2:**
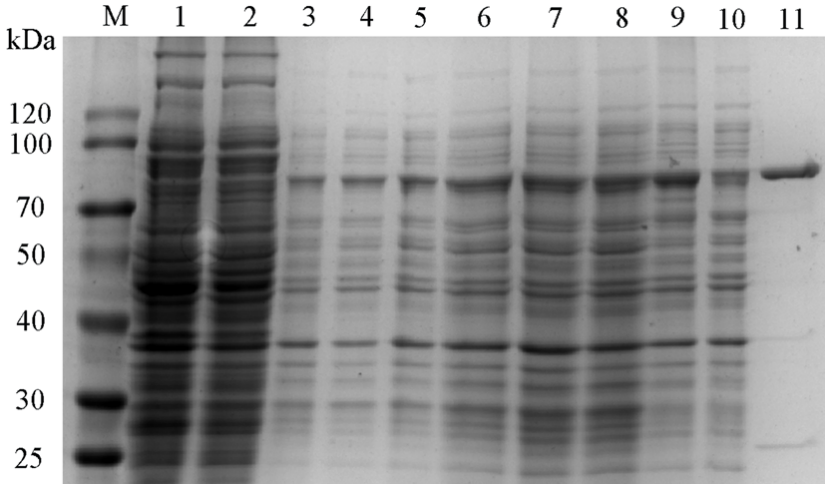
SDS-PAGE analysis of SlyUGT expressed in *E. coli* BL21 (DE3). Lane M: protein molecular mass marker; lane 1: the total protein of *E. coli* BL21 (DE3) harboring pGEX-2T; lane 2: the soluble protein of *E. coli* BL21 (DE3) harboring pGEX- 2T; lane 3, 5, 7, 9: the total protein of *E. coli* BL21 (DE3) harboring PGEX-SlyUGT at 20°C, 25°C, 30°C, and 37°C, respectively; lane 4, 6, 8, 10: the soluble protein of *E. coli* BL21 (DE3) harboring PGEX-SlyUGT at 20°C, 25°C, 30°C, and 37°C, respectively; lane 11: SlyUGT purified by GSTSep Glutathione Agarose Resin GST affinity.

The expression of plant-derived genes in *E. coli* often leads to inclusion bodies. Therefore, firstly, the effects of different induction temperatures on SlyUGT production were studied. As shown in Fig. 1A, the optimal induction temperature is 30°C. It can be seen from the SDS-PAGE protein gel (Fig. 2) that the expression of SlyUGT was overexpressed at 20°C, 25°C, 30°C and 37°C. Among them, the expression of protein was the highest at 37°C, but most of it existed in the form of inclusion bodies. However, the expression of SlyUGT at 20°C, 25°C and 30°C was basically soluble, and the expression level is the highest at 30°C. This result explains why the SlyUGT activity induced at 30°C is the highest. The concentration of inducer has great influence on the production of recombinant protein. Therefore, the effects of different IPTG concentrations on the production of SlyUGT by recombinant strain were studied at 30°C. The results showed that the optimum IPTG concentration was 0.1 mM (Fig. 1B), and the yield of SlyUGT was 4.5 times that without inducer. The optimal OD600 is 1.8 (Fig. 1C), which indicates that the optimal balance between bacterial growth and protein expression is achieved under this condition. In addition, the maximum enzyme activity was observed at 36 h after induction (Fig. 1D). It may be that the enzyme began to be degraded by protease after 36 h of induction. Under the optimum culture conditions, the maximum enzyme activity of the target protein SlyUGT was 29.48 mU/ml.

### Purification of SlyUGT

SlyUGT was purified by GSTSep Glutathione Agarose Resin GST affinity column, and the yield reached 53.9%. Its specific enzyme activity was 16.1 times higher than that of the crude enzyme, reaching 20.9 mU/mg (Table 1). Purified SlyUGT was detected by SDS-PAGE protein gel, and the results showed that its molecular mass was about 78.5 kDa (Fig. 2, Lane 11).

### Effect of Temperature and pH on Activity and Stability of SlyUGT

**Fig. 3:**
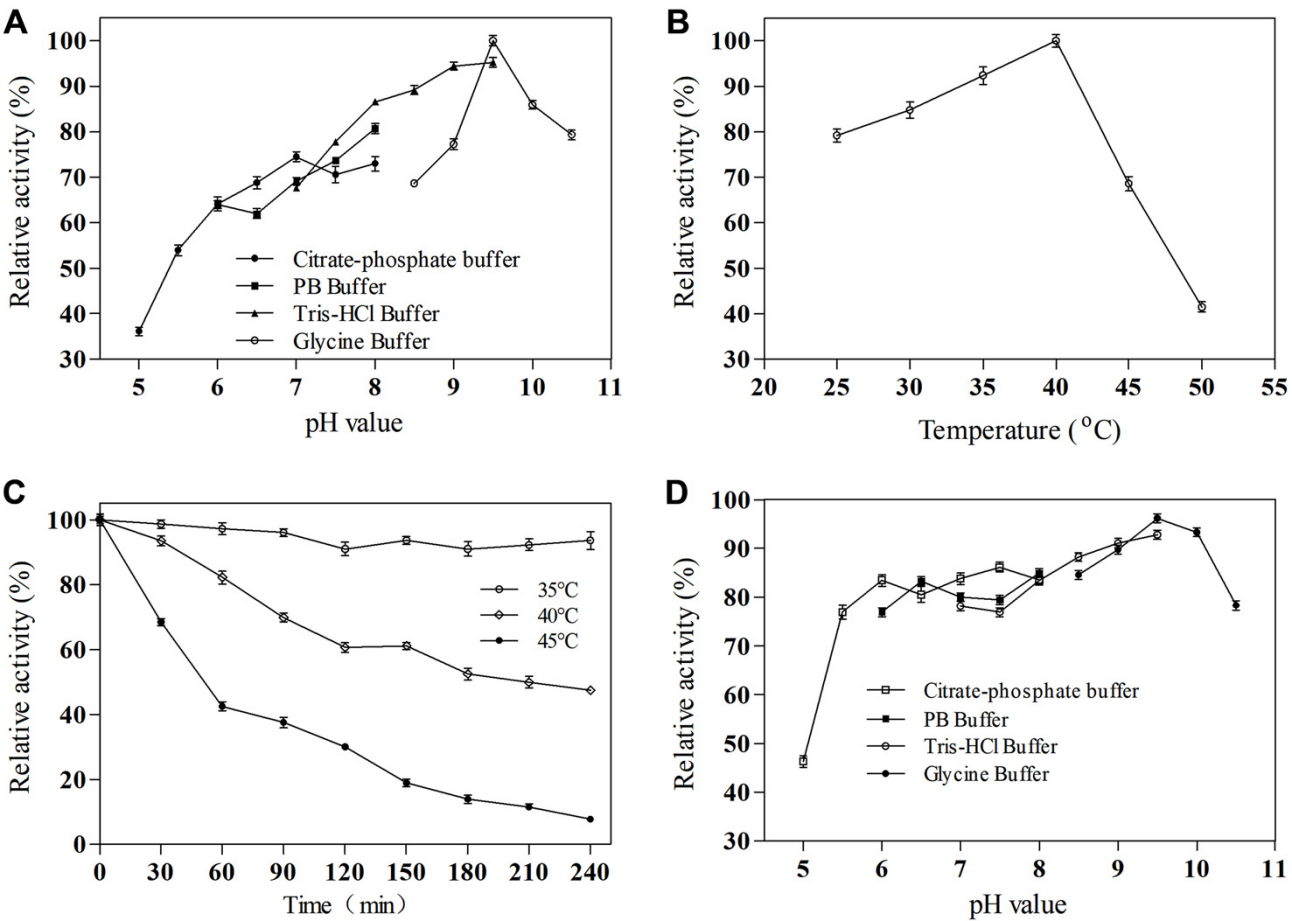
The effects of pH and temperature on the activity and stability of SlyUGT. (**A**) pH. (**B**) Temperature. (**C**) thermal stability. (**D**) pH stability; The initial activity was defined as 100%. These activities were expressed as relative values. Data represent the means of three experiments, and error bars represent the standard deviation.

pH and temperature are crucial factors in any enzyme catalyzed reaction. Therefore, the effects of pH and temperature on the purified SlyUGT activity were studied with *p*HBA as the substrate. As shown in Fig. 3A, the optimal pH of SlyUGT was determined to be 9.5 with glycine buffer, which was 5% higher than that in Tris-HCl buffer. This phenomenon also appears in other glycosyltransferase research reports [16, 17]. The results showed that the enzyme was an alkaline enzyme. Meanwhile, the SlyUGT maintains a high activity in the pH range of 5.5 to 10.5 (Fig. 3A), which indicates that the enzyme has a very wide pH application range. In addition, the relative enzyme activity of SlyUGT was the highest at 40°C (Fig. 3B), and the maximum enzyme activity can be maintained above 60% in the range of 25-45°C.

The thermal and pH stability of enzyme are also very important parameters in industrial application. Therefore, the thermal stability of SlyUGT at 35°C, 40°C, and 45°C was measured. The results showed that SlyUGT exhibited excellent thermal stability at 35°C, and the half-life of enzyme activity was about 210 min and 45 min at 40°C and 45°C (Fig. 3C), respectively. The pH stability of SlyUGT was further studied. As shown in Fig. 3D, SlyUGT had good stability in the buffer with pH of 5.5-10.5, and the relative enzyme activity remained above 70%.

### Effect of Metal Ions and Chemical Reagents on SlyUGT Activity

Common metal ions and chemical reagents were selected to determine the effect on SlyUGT activity, and their final concentrations in the reaction system were controlled to be 1 mM. As shown in Table 2, Cu^2+^, Hg^2+^ and Co^2+^ were found to completely inhibit the enzyme activity of SlyUGT, Similarly, glycosyltransferase from *Gentiana triflora* has the same result [18]. In addition, Zn^2+^, Fe^2+^, and Fe^3+^ can significantly reduce the enzyme activity of SlyUGT, and other metal ions can also inhibit the activity of SlyUGT. Moreover, EDTA, a metal chelating agent, have no significant effect on SlyUGT activity, indicating that free metal may not be important for maintaining the three-dimensional structure [19]. DTT, a sulfhydryl inhibitor, significantly reduced the SlyUGT activity, indicating that sulfhydryl groups may exist in the active center of SlyUGT [20].

### Kinetic Parameter of SlyUGT

**Fig. 4:**
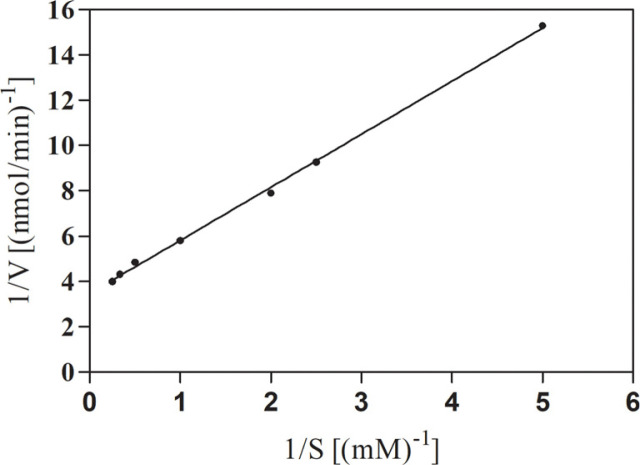
Lineweaver-Burk plot of SlyUGT activity with *p*HBA concentration.

Michaelis-Menten kinetics can elucidate the rate of an enzyme-catalyzed reaction based on the concentration of the enzyme and its substrate [21]. The values for kinetic constants of SlyUGT were measured from the double reciprocal Lineweaver-Burk plot. The values of K_M_ and V_max_ were found to be 0.65 mM and 74.60 mU/mg, respectively (Fig. 4). Consequently, the recombinant SlyUGT had a good affinity for *p*HBA.

### Effects of DMSO and UDP Concentration on the SlyUGT Activity

**Fig. 5:**
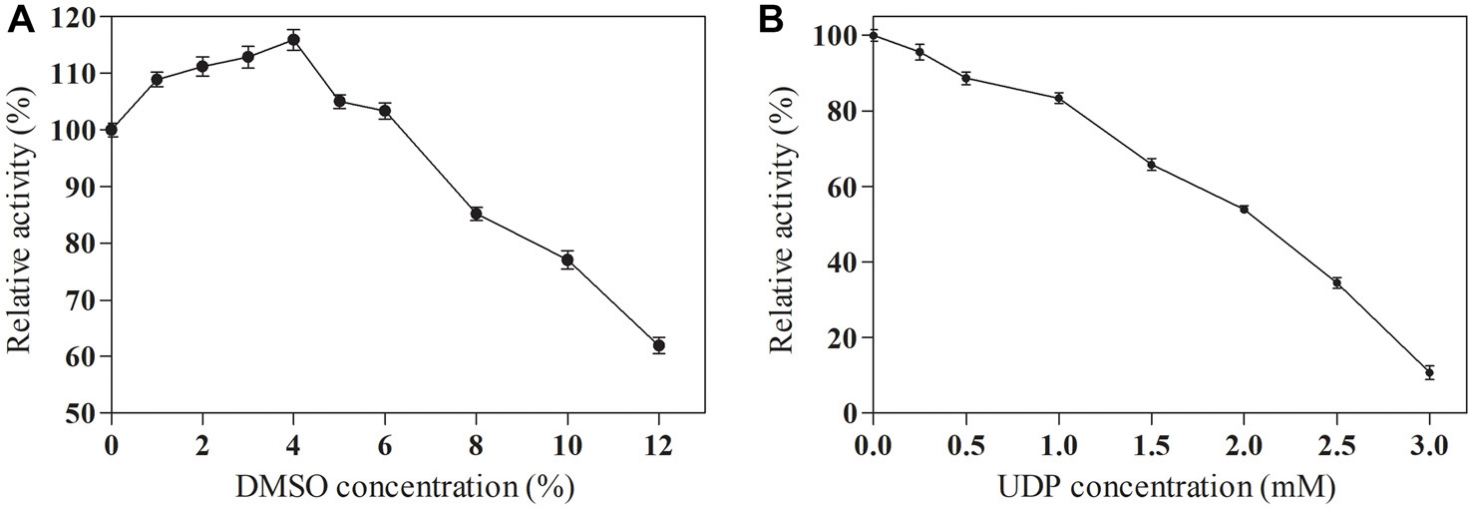
The effects of DMSO (A) and UDP (B) on the activity of the recombinant SlyUGT. The initial activity was defined as 100%. These activities were expressed as relative values. Data represent the means of three experiments, and error bars represent the standard deviation.

DMSO is toxic to enzyme and affects enzyme activity. Therefore, we studied the effects of different concentrations of DMSO on enzyme activity. As shown in Fig. 5A, in the range of DMSO concentration from 1%to 6%, the SlyUGT activity increased slightly. When DMSO concentration was 4%, the enzyme activity reached the highest, which increased by 16%. However, when DMSO concentration was higher than 6%, the enzyme activity decreased significantly. This has the same trend as the research results of Pei *et al*. [18]. Therefore, 4% DMSO will be used in subsequent experiments. In addition, the concentration of UDP affects the activity of glycosyltransferase [14, 18]. Therefore, we also studied the effects of different UDP concentrations on enzyme activity. As shown in Fig. 5B, when the concentration of UDP was in the range of 0-1.0 mM, the SlyUGT activity can still maintain more than 80% of the initial enzyme activity. However, when the concentration of UDP was higher than 1.0 mM, the enzyme activity decreased obviously. This has the same trend as the research results of Chen *et al*. [22]. Therefore, the concentration of UDP should be controlled to be less than 1.0 mM in the follow-up experiments, which will help SlyUGT maintain high enzyme activity, thus improving the transformation efficiency and product yield.

### Optimization of Gastrodin Biosynthesis Conditions

**Fig. 6:**
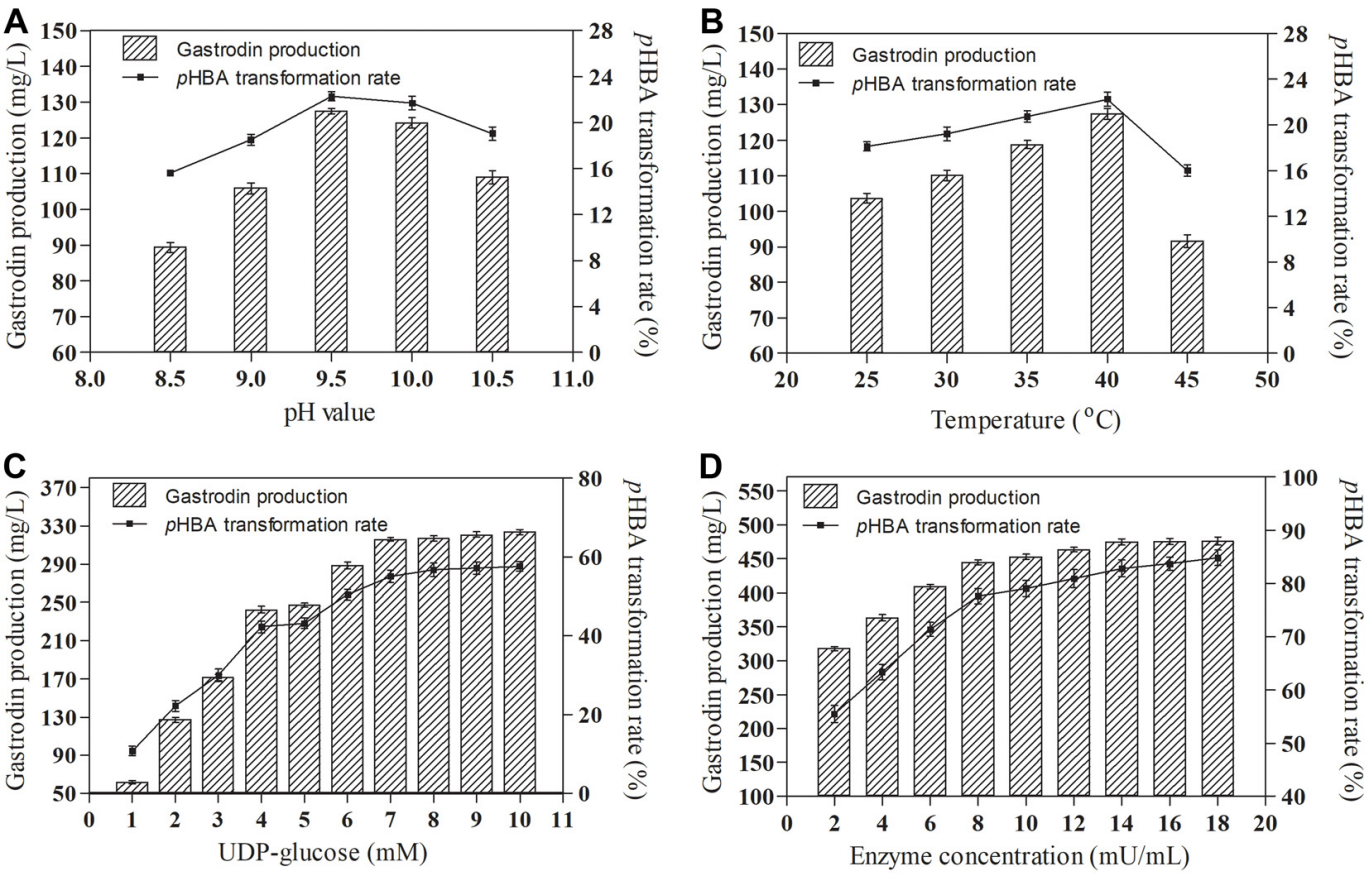
Optimization of biosynthesis conditions for gastrodin production by SlyUGT. Effects of pH (**A**) temperature (**B**) UDP-glucose concentration (**C**) SlyUGT concentration (**D**) on the gastrodin production and *p*HBA transformation rate. Data represent the means of three experiments, and error bars represent the standard deviation.

Since pH and temperature have important effects on enzyme activity, the effects of pH and temperature on gastrodin biosynthesis have been studied. As shown in Fig. 6A, the highest gastrodin production and *p*HBA transformation rate was 124.32 mg/l and 21.71% at pH9.5 respectively. This is because the optimum pH of SlyUGT is 9.5, and its stability is excellent at pH9.5. In addition, the gastrodin production and the conversion rate of *p*HBA were the highest at 40°C, reaching 127.41 mg/l and 22.25% respectively (Fig. 6B). This is because the optimum temperature of SlyUGT is 40°C, and the thermal stability is good at this temperature. UDP-glucose plays an important role as a sugar donor in the gastrodin biosynthesis. Therefore, the effects of different UDP-glucose concentrations on gastrodin biosynthesis were studied. When the UDP-glucose concentration was in the range of 1-7 mM, the gastrodin production and the conversion rate of *p*HBA were significantly improved, and the gastrodin production and *p*HBA conversion rate reached 315.66 mg/l and 55.13% respectively (Fig. 6C). However, when the UDP-glucose concentration was higher than 7 mM, the increase of gastrodin production and *p*HBA conversion rate was very slow (Fig. 6C). Therefore, the most suitable UDP-glucose concentration was 7 mM. Furthermore, we also studied the effects of different enzyme concentrations on gastrodin production and *p*HBA conversion rate. As shown in Fig. 6D, the gastrodin production and the *p*HBA conversion rate increased with the increase of enzyme concentration, and the optimal enzyme concentration was 14 mU/ml. At this time, the gastrodin production and the *p*HBA conversion rate reached 474.65 mg/l and 82.90% respectively.

### The Time Courses of Gastrodin Production

**Fig. 7:**
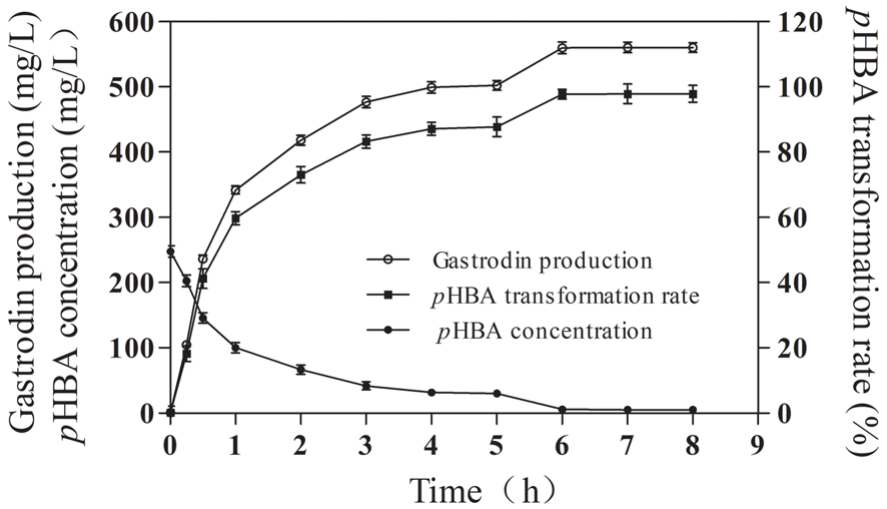
Time courses of gastrodin production, *p*HBA transformation rate, and *p*HBA concentration in reaction system. Data represent the means of three experiments, and error bars represent the standard deviation.

Based on the above-mentioned optimum conditions for biosynthesis of gastrodin, the changes of gastrodin production, *p*HBA conversion rate and *p*HBA concentration with reaction time were studied. Within 0.25 h after the start of biosynthesis, the specific productivity was 422.16 mg/l/h (Fig. 7). With the progress of the reaction, the specific productivity gradually increased, and its value was 526.80 mg/l/h within the reaction time of 0.25-0.5 h (Fig. 6). However, when the reaction time is more than 0.5 h, the specific productivity gradually decreases, reaching 210.88 mg/l/h within the reaction time of 0.5-1 h. In addition, after 1 h of reaction, the specific productivity decreased rapidly, reaching 67.44 mg/l/h in 1-3 h and 27.7 mg/l/h in 3-6 h. After 6 h, the gastrodin production was the highest, reaching 559.83 mg/l, and the molar conversion rate of *p*HBA reached 97.78%. Cui *et al*. studied the in vitro biosynthesis of gastrodin by itUGT from *Indigofera tinctoria*. The highest production and molar conversion rate were 535 mg/l and 93%, respectively [13], which were lower than those of SlyUGT. In addition, Xia *et al*. studied the in vitro biosynthesis of gastrodin by AtUGT from *Arabidopsis thaliana*, and the highest production reached 2.67 g/l [23], which is the highest in vitro biosynthesis of gastrodin at present. However, 2 mg of AtUGT enzyme protein was used in the reaction, so the ratio of gastrodin production to protein input was 1.13 g/mg. The biosynthesis of gastrodin by SlyUGT can reach 8.36 g/mg, which is 7.4 times that of AtUGT. Guo *et al*. can biosynthesize gastrodin in vitro by using RrUGT from *Rhodiola rosea* [24], but the concentration of *p*HBA was only 0.5 mM. Therefore, SlyUGT has obvious advantages in the biosynthesis of gastrodin in vitro.

In this study, UDP-glycosyltransferase from *Solanum lycopersicum* (SlyUGT) was overexpressed in *E. coli* BL21(DE3). The optimum temperature was 40°C, and it exhibited excellent thermal stability at 35°C and 40°C. In particular, the optimum pH of SlyUGT was 9.5, and it was an alkali-tolerant enzyme with excellent pH stability at pH5.5-10.5. The values of K_M_ and V_max_ were found to be 0.65 mM and 74.60 mU/mg respectively, and the enzyme has certain tolerance to DMSO and UDP. Under the optimal biosynthesis conditions, the gastrodin production reached 559.83 mg/l, and the corresponding molar conversion rate reached 97.82%, which is the highest gastrodin production in vitro enzymatic biosynthesis so far. The results showed that SlyUGT has potential application value in the preparation of gastrodin.

## Figures and Tables

**Table 1:** Purification process for the recombinant protein SlyUGT.

Purification step	Total protein (mg)	Total activity (mU)	Specific activity (mU/mg)	Yield (%)	Purification factor (fold)
Crude extract	3403.8	4425.0	1.3	100.0	1.0
GSTSep Glutathione Agarose Resin GST affinity.	114.1	2385.1	20.9	53.9	16.1

**Table 2:** Effects of metal cations and reagents on SlyUGT Activity.

Cation and reagent^a^	Relative activity (Mean% ± SD)	Cation and reagent^a^	Relative activity (Mean% ± SD)^a^
Control	100.0 ± 0.4	Ca^2+^	68.4 ± 0.8
Li^+^	77.4 ± 0.3	Zn^2+^	44.6 ± 0.2
K^+^	69.3 ± 0.2	Ba^2+^	75.8 ± 0.4
Na^+^	65.0 ± 0.3	Fe^2+^	16.3 ± 0.2
NH_4_^+^	72.3 ± 0.4	Hg^2+^	0 ± 0
Mn^2+^	92.4 ± 0.6	Fe^3+^	12.4 ± 0.3
Mg^2+^	99.7 ± 0.6	Al^3+^	66.4 ± 0.7
Cu^2+^	0 ± 0	EDTA	87.7 ± 0.6
Co^2+^	0 ± 0	DTT	53.4 ± 0.4

Values shown are the mean of duplicate experiments, and the SD represents the standard deviation.

^a^Final concentration of substrate was 1.0 mM
